# 51-Year-Old Male with Back Pain, Groin Pain, and a Rash

**DOI:** 10.5811/cpcem.41530

**Published:** 2025-07-14

**Authors:** Lorado Mhonda, Bobbi-Jo Lowie, Laura J. Bontempo, T. Andrew Windsor

**Affiliations:** * University of Maryland Medical Center, Department of Emergency Medicine, Baltimore, Maryland; † University of Maryland School of Medicine, Department of Emergency Medicine, Baltimore, Maryland

## Abstract

A 51-year-old male presented to the emergency department with back pain, bilateral groin pain, and bilateral leg numbness for four days. He was hypothermic, tachycardic, tachypneic, and hypotensive on presentation. A diffuse purpuric rash with bullae and desquamation was noted on exam. This case explores the differential diagnosis and evaluation of an ill patient who presented with an impressive rash.

## CASE PRESENTATION (DR. MHONDA)

A 51-year-old male presented to the emergency department (ED) with complaint of back pain, bilateral groin pain, and bilateral leg numbness. The patient reported that his back pain started four days after lifting his father. He described the pain as a constant burning sensation and muscle pain that radiated to his bilateral lower extremities and rated it a 10/10 in severity. He also reported a loss of balance, inability to urinate, fecal incontinence, fevers, and a dark-colored rash with blisters on his legs and groin. He reported that his legs felt cold. He denied any headache, dizziness, or neck pain.

On chart review of a prior healthcare visit from several years prior, the patient’s past medical history was significant for HIV, although he reported being unaware of this diagnosis. The patient’s medications were listed as darunavir, elvitegravir/cobicistat/emtricitabine/tenofovir, and maraviroc, but he reported he was not taking any of those medications. He denied any history of cancer or recent infections. His surgical history was significant for an exploratory laparotomy for an abdominal gunshot wound, and his social history included frequent inhaled marijuana and phencyclidine (PCP) use, but he denied any history of intravenous (IV) drug use. No prior laboratory tests were available in the electronic health record to review. He denied any known allergies.

The patient’s vitals were significant for hypothermia at 92.8 °Fahrenheit/33.8 °Celsius, tachypnea at 28 breaths per minute, tachycardia at 117 beats per minute, and hypotension at 90/62 millimeters of mercury. His oxygen saturation was 98% on room air. He weighed 73.4 kilograms with a body mass index of 29 (normal 18.5–25). He appeared ill and in acute distress. He was awake, alert, and oriented to person, place, and time with no cranial nerve deficits. His head was normocephalic and atraumatic. His neck was non-tender with full range of motion and no stiffness or rigidity. Pupils were equal, round, and reactive to light, and extraocular movements were intact. His oropharynx and ears were without erythema, edema, or lesions. On auscultation of the heart, the patient was noted to have a tachycardic regular rhythm with no rubs, murmurs, or gallops. His peripheral pulses were 2+ bilaterally. Lungs were clear to auscultation with no wheezing or rales noted and the patient was tachypneic but not in respiratory distress.

**Image 1:**
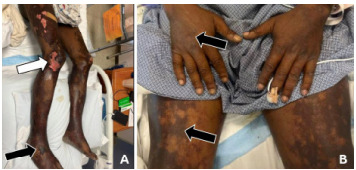
Rash on a 51-year-old male who presented with back pain, lower extremity numbness, and a progressive purpuric rash. (A) Desquamation (white arrow), non-blanching purpura and hemorrhagic bullae (black arrow) on the bilateral lower extremities. (B) Non-blanching purpura (black arrows) on the bilateral dorsal hands and bilateral legs.

The patient’s abdomen was diffusely tender on palpation with voluntary guarding and no rebound or distention. He did not have any costovertebral angle tenderness. He was tender to palpation in the bilateral groin and scrotal regions with purpura, desquamation, warmth, and edema. No lymphadenopathy was noted. His rectal tone was intact with no evidence of urinary retention on bladder ultrasound. His bilateral lower extremities and back were tender on palpation with full range of motion, 5/5 strength, and no signs of trauma or edema. Sensation was diffusely decreased in the bilateral lower extremities. Non-blanching purpura was noted on his lower extremities and dorsal aspect of his hands. Bullae and desquamation were also present on his lower extremities with a positive Asboe-Hansen sign and negative Nikolsky sign (Image 1). There was no rash involvement of his face, torso, or mucosal membrane.

**Image 2:**
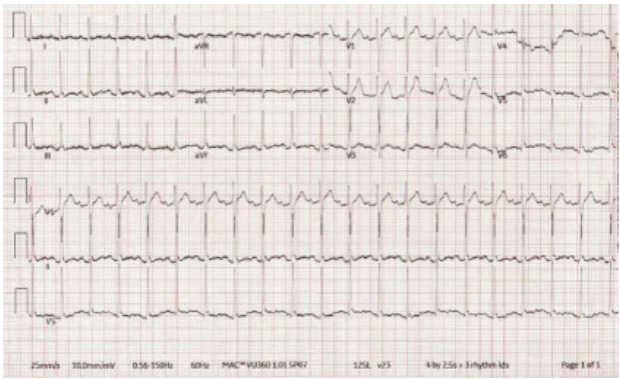
Electrocardiogram of a 51-year-old male who presented with back pain, lower extremity numbness, and a progressive purpuric rash.

**Image 3:**
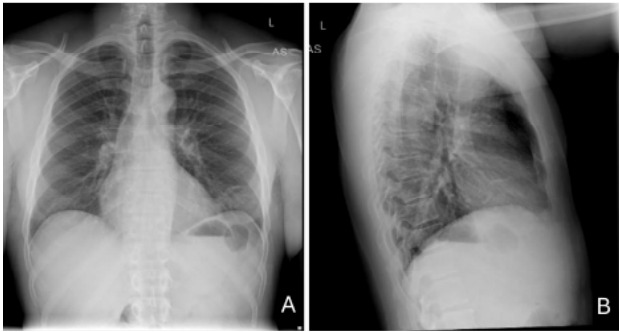
Posterior-anterior (A) and lateral (B) chest radiographs of a 51-year-old male who presented with back pain, lower extremity numbness, and a progressive purpuric rash.

Laboratory studies (Table), blood cultures, and an electrocardiogram (ECG) (Image 2) were obtained. Chest radiography (Image 3) and computed tomography (CT) with contrast of the cervical, thoracic, and lumbar spine, and of the chest, abdomen, and pelvis were also completed but did not demonstrate any acute abnormalities to explain the patient’s symptoms. Bilateral lower extremity ankle brachial indices (ABI) were normal. Ultimately, a diagnostic test was performed that confirmed the diagnosis.

## CASE DISCUSSION (DR. LOWIE)

This is a case of a 51-year-old male who presented to the ED with an initial chief complaint of back pain, groin pain, and bilateral leg numbness. His back pain had started four days prior to his presentation, was initially attributed to lifting his father, and described as a burning sensation that radiated to his groin and legs bilaterally. He went on to report numbness in his legs, loss of balance, urinary retention, and fecal incontinence. Notably, he had also reported fevers and a dark rash with blisters on his legs and groin. He had a documented history of HIV but had never started treatment as he was apparently not aware of this diagnosis. He denied any history of IV drug use, nicotine use, or alcohol use, but frequently used marijuana and PCP.

His triage vitals were almost all abnormal as he was tachycardic, hypothermic, borderline hypotensive, and tachypneic. His examination revealed many abnormalities, and he was noted to be in distress with diffuse abdominal guarding and a desquamating purpuric rash on the bilateral groin, scrotum, and legs with a positive Asboe-Hansen sign. He also had purpura of the bilateral dorsal hands. Interestingly, while the patient reported decreased sensation to touch of his extremities, he did not have any focal neurologic deficits; he had normal rectal tone and no urinary retention on point-of-care ultrasound. Some pertinent negatives from his examination were that he had no lesions in the oropharynx and a negative Nikolsky sign.

An extensive workup was initiated by the team including blood, urine, and multiple imaging studies. Some key findings from this workup included elevated blood urea nitrogen and creatinine, elevated creatine kinase levels, a bandemia, thrombocytopenia, and a D-dimer level that was greater than the upper limit that the laboratory could report. The ECG showed sinus tachycardia but was otherwise non-diagnostic. He had a chest radiograph, which did not reveal any infiltrates, pneumothorax, or effusions. Computed tomography was apparently not revealing of surgical pathology, and the normal ABI testing lessened the likelihood of acute vascular compromise.

The differential diagnosis for this patient is quite broad given the many initial presenting complaints, which seem to be pulling in many different directions. Is this a case where Occam’s razor applies and all symptoms can be explained by one diagnosis, or Hickam’s dictum, where many different diseases may be coinciding all at once? Perhaps there are some red herrings here distracting from the true problem. At first glance, the chief complaint seems to be pointing toward spinal cord pathology or a primary neurologic complaint given the back pain, numbness, bladder and bowel complaints. However, we quickly learn of this patient’s normal motor examination followed by the finding of purpura, which cannot be ignored.

Purpura is a tangible starting point when attempting to determine this patient’s diagnosis. The broad categories where purpura can be found include trauma, infections, vasculitis, drug-induced, vitamin deficiencies, collagen disorders, pigmented purpuric dermatosis, and disorders of hemostasis.^1^ Trauma can easily be ruled out here as the patient had no reports or evidence of trauma, and the examination also supports no findings of traumatic injury. Next, we can easily eliminate drug-induced purpura as the patient does not seem to be taking any medications, and marijuana and PCP are not considered culprits of drug-induced purpura. Vitamin C deficiency is also unlikely in this patient as this is exceedingly rare in developed countries, and the patient has systemic illness not generally seen in vitamin C deficiency. Collagen disorders like Ehlers-Danlos syndrome include joint hypermobility and hyperextensibility, which are not present in this patient. Pigmented purpuric dermatosis is a capillaritis that can cause purpura localized to the lower extremities; however, it is usually non-painful and not associated with systemic illness.

While it seems that we have eliminated many diagnoses quickly, there are still more broad categories to discuss, including disorders of hemostasis. This includes the subcategories of thrombocytopenia, platelet function abnormalities, clotting factor deficiencies, and disseminated intravascular coagulopathy (DIC). The patient does have profound thrombocytopenia, and any hereditary cause of thrombocytopenia could be easily eliminated as most should have manifested before the patient reached adulthood. Similarly, vitamin deficiences induced by use of drugs or alcohol could again be eliminated from the differential. You may see thrombocytopenia in patients with chronic alcohol use, but the patient has no history of alcohol use. He does report frequent marijuana and PCP use; however, neither marijuana nor PCP are known to result in thrombocytopenia.

Some notable diagnoses that require further thought include immune thrombocytopenia (ITP), thrombotic thrombocytopenic purpura (TTP), paroxysmal nocturnal hemoglobinuria, and hemolytic uremic syndrome (HUS). While ITP is common in children, it can also occur in patients with HIV. It classically causes isolated thrombocytopenia, which we do see in this patient, but it does not explain the renal dysfunction and what appears to be systemic illness. Both TTP and HUS cause thrombocytopenia and renal failure but should also result in hemolysis and anemia, which is not seen in this patient. Paroxysmal nocturnal hemoglobinuria can cause thrombocytopenia with renal dysfunction but also causes anemia, which again, the patient does not have. Platelet function abnormalities and clotting factor deficiencies could also result in purpura; however, they are often inherited disorders or secondary to drugs or other underlying processes such as infection, trauma, or uremia. Alone, these do not explain this patient’s presentation. The same goes for DIC, which is often seen because of an underlying process and is not, in fact, a diagnosis itself. Additionally, the laboratory workup is not consistent with a diagnosis of DIC due to the normal fibrinogen and partial thromboplastic time values, both of which are typically low.

Next, we should consider vasculitis as a cause of purpura, which entails another long list of diagnoses. The broad categories of vasculitis include small, medium, and large vessel vasculitis, immune complex, variable vessel, and single-organ vasculitis. The medium and large vessel vasculitides including Kawasaki disease, Takayasu arteritis, giant cell arteritis (GCA), and polyarteritis nodosa (PAN) can be eliminated: Takayasu usually occurs before the age of 30; Kawasaki is seen in children; GCA usually results in headache and jaw claudication; and PAN results in erythematous nodules, different from the purpura seen in this patient. Of the small vessel vasculitides, a few need to be considered carefully. Microscopic polyangiitis and granulomatosis with polyangiitis are interesting to consider in this patient as they can result in renal dysfunction as well as arthralgias and paresthesias of the hands and feet, in addition to the skin manifestations of purpura. However, they generally include ear, nose, throat, and even pulmonary findings which the patient did not have.

Immunoglobulin A (IgA) vasculitis, formerly known as Henoch-Schonlein purpura, also results in purpura of the lower extremities with renal dysfunction and can also have gastrointestinal manifestations causing abdominal pain. However, one key feature making this diagnosis less likely is that the purpura in IgA vasculitis is often not painful. Finally, cryoglobulinemia can similarly be considered given his paresthesias, purpura, and history of HIV, but as the findings did not worsen with cold temperatures and there was no foot or wrist drop on exam, this diagnosis is also unlikely.

This patient has a history of HIV that has gone untreated for an unknown length of time, putting him at risk for certain types of malignancy including Kaposi sarcoma and non-Hodgkin lymphoma. Kaposi sarcoma is defined by a purplish-brown lesion on the skin, but it also includes the mucosal surfaces. No mucosal findings are reported in this patient and, additionally, the physical exam revealed the lesions to be Absoe-Hansen positive, which would not be the case in Kaposi sarcoma. Lymphoma is also very unlikely as this patient’s underlying diagnosis as the presentation was acute, and there was no reported lymphadenopathy or symptoms reported that were classic to this presentation.

Finally, we must think about infection as the cause of purpura and this patient’s underlying presentation, especially with his untreated HIV. He presented with many vital sign abnormalities concerning for infection. When it comes to infection in this patient, he is absolutely at risk both for opportunistic infections and severe or disseminated infection involving common pathogens. When considering the patient’s rash and overall presentation, purpura fulminans (PF) is fitting; it is usually a result of underlying severe infection or sepsis in adults. Purpura fulminans is a syndrome of microvascular thrombosis that results from an acquired protein-C deficiency that causes skin necrosis. It can be associated with end-organ damage and lab results often include the renal dysfunction also seen in this patient, as PF can act as a small-medium vessel vasculitis. Additionally, the lab results support this diagnosis including thrombocytopenia and a significantly elevated D-dimer. Coagulation studies can be normal or elevated and, if done, a protein-C level will be low. Interestingly, fibrinogen levels (normal in this patient) can be normal because while infection can increase levels, the microthrombosis may lower it. Infection is also supported by the bandemia seen on the automated differential. While bandemia is not always a result of infection, a bandemia of 14% is certainly concerning for infection.

At this point a decision must be made on what is the most likely underlying pathogen. There is an extensive list of possible infections. Looking at the data on PF, one of the most common culprits is *Neisseria meningitidis*. In fact, up to 20% of patients with *N meningitidis* develop PF.^2^ The patient is not presenting with typical signs and symptoms of meningitis in this case but rather of sepsis and meningococcemia. Therefore, after careful consideration of the facts of this case, my test of choice would be a blood culture revealing my final diagnosis of meningococcemia, resulting in PF.

## CASE OUTCOME (DR. MHONDA)

Blood cultures obtained in the ED demonstrated *N meningitidis* bacteremia. Dermatology was consulted for the extensive purpuric rash on his bilateral lower extremities and groin, concerning for PF. Punch biopsy was completed, and it showed extensive epidermal necrosis suggestive of thrombotic vasculopathy. The bullae continued to worsen with skin denuding and sloughing in the bilateral lower extremities, requiring aggressive rehydration and skin care. During his admission, the patient developed worsening abdominal pain and distension. A repeat CT of the abdomen and pelvis was completed, and it showed findings suggestive of spontaneous bacterial peritonitis. The patient also spontaneously developed dry gangrene of multiple toes in his bilateral feet. Vascular surgery recommended no acute intervention in the setting of intact pedal pulses and an unremarkable ABI and felt this was due to small vessel ischemia. During his admission, he was treated with broad spectrum antibiotics, narrowed based on culture and susceptibilities. He was also restarted on his HIV antiretroviral medication leading to improvement in his cluster of differentiation 4 (CD4) cells and viral load and fortunately was able to be discharged after 23 days of hospitalization with primary care, wound care, and podiatry outpatient follow-up.

## RESIDENT DISCUSSION (DR. MHONDA)

Meningococcal septicemia is a bloodstream infection caused by *N meningitidis* bacteria, which is an encapsulated Gram-negative diplococcus transmitted through respiratory droplets or secretions. Initial infection results from direct contact with respiratory secretions. The bacteria colonizes the respiratory tract and invades the nasopharyngeal epithelium. The meningococcal bacteria successfully adheres, invades, and proliferates due to its structural components that protect against phagocytosis and lysis. The adhesion of the bacteria to the epithelial and endothelial cells activates the innate immune system.^3^

The activation of the immune system leads to the release of multiple inflammatory mediators. These mediators activate multiple pathways including the coagulation cascade, the leukotriene, prostaglandin, and complement pathways. These subsequently lead to increased capillary permeability, pathologic vasoconstriction and vasodilation, coagulopathy and severe myocardial dysfunction. These series of events are responsible for the development of shock and end-organ failure.^3^

Risk factors for meningococcal septicemia include conditions that weaken the immune system’s response to the bacteria. These include complement deficiencies and inhibitors, HIV, and functional or anatomic asplenia.^4^ Individuals who are younger than one year of age are at increased risk as they have not developed an appropriate immune system to adequately fight against the bacteria.^4^ Smoking is another risk factor, as it results in the destruction of the initial nasopharyngeal epithelium protective barrier against bacterial invasion.^5^ Individuals living in crowded conditions, including college students, military recruits, those of low socioeconomic status, and travelers to the “meningitis belt” in Sub-Saharan Africa, are also at an increased risk.^6^

Meningococcal septicemia initially presents as high fevers with shaking chills, severe myalgias, tachycardia, normotension, and cold extremities. The patient then develops petechiae, which transforms into diffuse PF and ultimately necrosis with gangrene. The changes can occur within hours. The vascular damage leads to hypotension, adrenal hemorrhage also known as Waterhouse-Friderichsen syndrome, cardiac failure, renal failure, and acute respiratory distress syndrome. 7

*Neisseria meningitidis* is diagnosed through clinical presentation, Gram stain, cultures or antigen detection, with the gold standard being cultures. Gram staining will show Gram-negative diplococci. Cultures can be obtained from the mucosa, cerebrospinal fluid (CSF), and blood but have variable sensitivity.^8^ Cerebrospinal fluid cultures have a 90% sensitivity, blood cultures a 40–75% sensitivity, and a combination of both has a 94% sensitivity.^3^ Antigen detection through deoxyribonucleic acid polymerase chain reaction of the CSF, plasma, or serum can also be completed with a sensitivity and specificity greater than 90% and has the advantage of being able to rapidly detect the organism.^3^

Electrolyte and metabolic derangements can also be present including hypoglycemia, hypokalemia, hypocalcemia, hypomagnesemia, hypophosphatemia, and metabolic acidosis. Patients can also be hematologically unstable with anemia and decreased protein C, fibrinogen, prothrombin, and coagulation factors (V, VII, and X).^2^ Primary treatment for meningococcal septicemia is intravenous (IV) ceftriaxone or cefotaxime.^8^ Alternative therapy includes IV penicillin G or IV ampicillin.^8^,^9^ Ideally, culture data should be obtained prior to initiating penicillin due to increased resistance. Patients can also be treated with chloramphenicol, but it is less effective when compared to the other antibiotics. Patients with severe allergies and unavailable antibiotic sensitivity information can be treated with IV meropenem instead.^9^ Chemoprophylaxis should be administered to close contacts of the patient. Prophylactic treatments include rifampin, ceftriaxone and ciprofloxacin.^8^

## FINAL DIAGNOSIS

Meningococcal septicemia

## KEY TEACHING POINTS

- Purpura fulminans in adults is most commonly due to severe infection.
- Meningococcal septicemia can rapidly progress from seemingly benign flu-like symptoms to multi-organ failure.
- Empiric treatment with IV antibiotics should be started as soon as meningococcal infection is suspected, even before confirmation, due to the rapid and life-threatening nature of the disease.

## Figures and Tables

**Table:** Initial laboratory results in a 51-year-old male with back pain, groin pain, and a rash.

Test Name	Patient Value	Reference Range
Complete Metabolic Panel		
Sodium	133 mmol/L	136 – 145 mmol/L
Potassium	3.9 mmol/L	3.5 – 5.1 mmol/L
Chloride	94 mmol/L	98 – 107 mmol/L
Bicarbonate	17 mmol/L	22 – 29 mmol/L
Blood urea nitrogen	46 mg/dL	6 – 23 mg/dL
Creatinine	4.3 mg/dL	0.7 – 1.2 mg/dL
Glucose	88 mg/dL	74 – 100 mg/dL
Calcium	9 mg/dL	8.6 – 10.2 mg/dL
Magnesium	1.6 mg/dL	1.6 – 2.6 mg/dL
Phosphorus	6 mg/dL	2.5 – 4.5 mg/dL
Aspartate aminotransferase	85 unit/L	5 – 40 unit/L
Alanine aminotransferase	26 unit/L	5 – 41 unit/L
Total Bilirubin	1 mg/dL	0.1 – 1 mg/dL
Alkaline phosphatase	76 unit/L	38 – 126 unit/L
Anion gap	21 mmol/L	8–18 mmol/L
Complete Blood Count		
White blood cells	4 K/mcL	4.5 – 11 K/mcL
Hemoglobin	14.1 g/dL	11.9 – 15.7 g/dL
Hematocrit	44.5%	35.0 – 45.0%
Platelets	23 K/mcL	153 – 367 K/mcL
Platelet estimate	Decreased	Normal
Platelet morphology	Normal	Normal
Neutrophil %	78%	40–60%
Bands	14%	0–3%
Lymphocytes %	3%	20–40%
Monocytes %	5%	2–8%
Coagulation		
PT	22.5 sec	12.0 – 15.0 sec
INR	2	<= 1.4
PTT	32.8 sec	22.0 – 36.0 sec
D-dimer	>20.00 mcg/mL FEU	0.27 – 0.50 mcg/mL FEU
Fibrinogen	539 mg/dL	160 – 600 mg/dL
Urinalysis		
Color	Dark Yellow	Yellow
Appearance	Turbid	Clear
Specific gravity	>= 1.030	1.002 – 1.030
pH	5	5.0 – 8.0
Glucose	Negative	Negative
Bilirubin	1+	Negative
Urobilinogen	1.0 EU/dL	0.2 EU/dL
Ketones	Trace	Negative
Blood	1+	Negative
Protein	3+	Negative
Nitrite	Negative	Negative
Leukocyte esterase	Trace	Negative
White blood cells	Too numerous to count	0–5/hpf
Red blood cells	11 – 20/hpf	0–5/hpf
Squam epithelial cells	Too numerous to count	0–2/hpf
Hyaline casts	Too numerous to count	0–2/hpf
Bacteria	Negative	Negative
Additional Labs		
HIV antigen/antibody	Reactive	Non-Reactive
HAV immunoglobulin M	Non-Reactive	Non-Reactive
HCV antibody	Non-Reactive	Non-Reactive
HBV surface antigen	Non-Reactive	Non-Reactive
HBV core immunoglobulin M	Non-Reactive	Non-Reactive
Rapid plasma reagin	Non-Reactive	Non-Reactive
SARS-CoV-2 (COVID-19 PCR) RNA	Not Detected	Not Detected
Influenza A	Not Detected	Not Detected
Influenza B	Not Detected	Not Detected
Respiratory syncytial virus	Not Detected	Not Detected
Folate	5.2 ng/mL	4.8 – 20.0 ng/mL
Vitamin B12	533 pg/mL	211 – 946 pg/mL
Lactate	5.8 mmol/L	0.5 – 2.2 mmol/L
Creatine kinase	1633 unit/L	39 – 308 unit/L
Myoglobin	1834 mg/mL	28 – 72 mg/mL

Abbreviations: *PT*, prothrombin time; *PTT*, partial thromboplastin time; *INR*, international normalized ratio; *HIV*, human immunodeficiency virus; *HAV*, hepatitis A virus; *HBV*, hepatitis B virus; *SARS-CoV-2*, severe acute respiratory syndrome coronavirus 2*; COVID-19*, coronavirus disease 2019*; RNA*, ribonucleic acid; *K*, thousands; *mcL*, microliter; *g*, grams; *dL*, deciliter; *mmol*, millimole; *L*, liter; *mg*, milligram; *ng*, nanogram; *pg*, picogram; *hpf*, high powered field; *sec*, seconds; *EU*, Ehrlich unit; *FEU*, fibrinogen equivalent units.
